# Wavelet scattering networks in deep learning for discovering protein markers in a cohort of Swedish rectal cancer patients

**DOI:** 10.1002/cam4.6672

**Published:** 2023-11-28

**Authors:** Tuan D. Pham, Xiao‐Feng Sun

**Affiliations:** ^1^ Barts and The London School of Medicine and Dentistry Queen Mary University of London Turner Street London UK; ^2^ Division of Oncology Department of Biomedical and Clinical Sciences Linkoping University Linkoping Sweden

**Keywords:** artificial intelligence, biomarkers, immunohistochemistry, rectal cancer

## Abstract

**Background:**

Cancer biomarkers play a pivotal role in the diagnosis, prognosis, and treatment response prediction of the disease. In this study, we analyzed the expression levels of RhoB and DNp73 proteins in rectal cancer, as captured in immunohistochemical images, to predict the 5‐year survival time of two patient groups: one with preoperative radiotherapy and one without.

**Methods:**

The utilization of deep convolutional neural networks in medical research, particularly in clinical cancer studies, has been gaining substantial attention. This success primarily stems from their ability to extract intricate image features that prove invaluable in machine learning. Another innovative method for extracting features at multiple levels is the wavelet‐scattering network. Our study combines the strengths of these two convolution‐based approaches to robustly extract image features related to protein expression.

**Results:**

The efficacy of our approach was evaluated across various tissue types, including tumor, biopsy, metastasis, and adjacent normal tissue. Statistical assessments demonstrated exceptional performance across a range of metrics, including prediction accuracy, classification accuracy, precision, and the area under the receiver operating characteristic curve.

**Conclusion:**

These results underscore the potential of dual convolutional learning to assist clinical researchers in the timely validation and discovery of cancer biomarkers.

## 1 INTRODUCTION

Cancer associated with the colon or rectum or colorectal cancer (CRC) constitutes high morbidity and mortality worldwide. CRC is a condition of colon or rectal cancer, depending on where the cancer begins in the colon or rectum, respectively. Colon cancer and rectal cancer are often grouped together known as CRC because these two cancers share many similar features, including symptoms, risk factors, and basic cell biology. Because cancer is a condition of genetic variations,^1^, ^2^ where the mutations can cause genes to function abnormally and result in alternative expression. Proteins, which are final products of gene expression, have influence on phenotypes and various biological processes. Hence, the capacity to discern the levels of protein expression holds clinical significance in the domain of cancer diagnosis, prognosis, treatment, and the anticipation of cancer recurrence.^1^, ^3^

Radiotherapy (RT) is a treatment for cancer. RT applies radiation to eliminate cancerous cells and shrink tumors. While RT does not consider the genetic makeup of tumors, it plays a significant role in cancer treatment and cost reduction within the health care system. A primary challenge in the use of RT, which may lead to its inappropriate application, is the absence of biomarkers for identifying cancer patients who could benefit from RT and respond favorably to the treatment.^4^ Because biomarkers serve as quantifiable biological indicators utilized in diagnosis, prognosis, and therapy; the exploration of cancer biomarkers holds immense importance, as they can be harnessed for precision or personalized cancer medicine. By offering insights into the individual biological mechanisms and the progression of cancer, these biomarkers empower clinicians to select effective therapeutic strategies.^5^, ^6^

Numerous endeavors have been made to identify biomarkers associated with various aspects of cancer.^7^, ^8^, ^9^, ^10^, ^11^, ^12^, ^13^ To cite a few examples: mutations in the BRAF, KRAS, and p53 genes have been implicated in CRC development^14^; the protein guggulsterone, a plant phytosteroid, has shown connections to inhibiting the growth of human CRC cells^15^; the expression of APRIL, BAFF, IL8, and MMP2 has been linked to the inflammatory microenvironment of CRC tumors^16^; multiple biomarkers have been explored in young CRC patients^17^; the evaluation of DNp73^18^ and RhoB^19^ expressions through immunohistochemistry (IHC) in rectal cancer tumor and biopsy samples; fecal microbial biomarkers have demonstrated potential for early CRC diagnosis^20^; and the gut microbiome, encompassing stool, blood, tissue, and bowel fluid samples, has been extensively investigated as a primary source of biomarkers for CRC screening.^21^

Being a novel dimension for biomedical research and health care, artificial intelligence (AI) and particularly convolutional neural networks (CNNs) known as deep learning are increasingly making themselves indispensable to the reality of precision or personalized medicine.^22^, ^23^, ^24^, ^25^ Furthermore, AI becomes a revolution in cancer study. For examples, AI has been applied as a powerful tool for precision histology,^26^ predicting unknown cancers,^27^ diagnosis and therapy,^28^ aiding histopathologists for improving clinical oncology,^29^ and cancer research.^30^

Among various convolutional network types, the wavelet‐scattering transform^31^, ^32^, ^33^ has appeared as a topic of growing interest within the signal processing and machine learning communities. This approach continues finding applications in diverse fields, including but not limited to neural disease classification,^34^ authentication of artworks,^35^ predictive indoor fingerprinting‐based localization,^36^ ECG beat classification,^37^ classification of alcoholic EEG signals,^38^ and magnetohydrodynamic simulations for pattern analysis.^39^

Based on the successful applications of convolutional methods in CNNs and wavelet scattering, this paper attempts, for the first time, to combine these two types of convolution‐based operations for extracting strongly differentiable features of protein‐expressed IHC images. Following the extraction of these robust features, a support vector machine (SVM) model is deployed to learn and predict the survival time for two patient cohorts with rectal cancer. These cohorts comprise patients who either received preoperative RT or did not, with the prediction goal being to differentiate between survival times of less than or more than 5 years.

## 2 MATERIALS AND METHODS

### 2.1 IHC imaging data

The study utilized a dataset consisting of IHC images collected from 136 patients who were enrolled in the randomized Swedish Rectal Cancer Trial conducted between 1987 and 1990.^40^ Out of these 136 patients, 77 did not receive RT, while the remaining 59 patients underwent RT. All patients were followed up for a period exceeding 5 years. It has been reported that there were no statistically significant differences (*p* > 0.05) between the two survival groups with respect to clinical and pathological characteristics, including factors such as sex, age, TNM (tumor‐node‐metastasis) stage, and degree of differentiation (see Tables S1 and S2 in^18^ provided in the Supporting Information section of a previous study,^18^ which presented the characteristics of patients and p‐values using the same IHC data).

Tissue microarray (TMA) slides were prepared from rectal cancer‐confirmed tissue samples, with validation by a pathologist. These samples were subsequently subjected to immuno‐histochemical staining, either using RhoB or DNp73 markers. The tissue specimens were initially fixed with formalin, embedded in paraffin, and fashioned into TMAs. TMA sections measuring 4 μm in thickness underwent deparaffinization through a series of treatments involving 100% xylene and ethanol, followed by antigen retrieval. Subsequently, the sections were subjected to overnight incubation with anti‐RhoB or anti‐DNP73 mouse monoclonal antibodies, followed by a 25‐minute incubation with a secondary antibody, Envision System Labelled Polymer‐HRP Anti‐Mouse. The sections were then exposed to Liquid DAB+ (Dako) and lightly counterstained with hematoxylin. Finally, the slides were digitally scanned using a Leica Aperio CS2 scanner.

To avoid the problem of data imbalance in machine learning and to fairly demonstrate the discovery of protein expressions, the numbers of IHC images of RhoB and DNp73 expressions for both survival times are designed to be equal by reducing the number of the images of the majority class to that of the minority class. For RhoB expression with the RT case, total samples of < and >5 year survival times for tumor = 106 (average of 1.80 samples per patient), biopsy = 102 (average of 1.73 samples per patient), metastasis = 20 (average of 0.34 sample per patient), and normal tissue adjacent to tumor = 58 (average of 0.98 sample per patient). For RhoB expression with the non‐RT case, total samples of < and >5‐year survival times for tumor = 132 (average of 1.71 samples per patient), biopsy = 90 (average of 1.17 samples per patient), metastasis = 20 (average of 0.26 sample per patient), and normal tissue adjacent to tumor = 76 (average of 0.99 sample per patient). Only IHC images of tumor and biopsy tissues are available for DNp73 expression. For DNp73 expression with the RT case, total samples of < and >5‐year survival times for tumor = 46 (average of 0.78 sample per patient), and biopsy = 22 (average of 0.37 sample per patient). For DNp73 expression with the non‐RT case, total samples of < and >5‐year survival times for tumor = 50 (average of 0.65 sample per patient), and biopsy = 28 (average of 0.36 sample per patient).

Both RhoB and DNp73 datasets were previously studied in,^18^, ^19^ respectively. Ten‐fold cross‐validation was used in this study for training and testing various machine‐learning models. This validation is particularly useful when the amount of data are limited. First, it divides the dataset into ten equal parts or “folds.” Each fold should contain approximately the same distribution of data points as the entire dataset. The next step involves a loop that iterates ten times. In each iteration, one of the 10 folds is set aside as the test set, and the remaining nine folds are used as the training set. A machine‐learning model is trained on the training set. The trained model is then used to make predictions on the test set.

### 2.2 Pretrained CNN models

Pretrained CNNs refer to CNN models that have been trained on large datasets for the task of image classification or related computer vision tasks before being used for other tasks. These pretrained models are a subset of transfer learning techniques in deep learning. How pretrained CNNs work is described as follows.

- Initial training: pretrained CNNs are initially trained on a massive dataset, such as ImageNet, which contains millions of labeled images across thousands of categories. During this training process, the CNN learns to recognize various features, patterns, and objects in images.
- Feature extraction: the layers of a CNN are composed of multiple convolutional, pooling, and fully connected layers. These layers capture hierarchical features from the input images, starting from low‐level edges and textures to higher level object parts and whole objects. The final layers typically represent class‐specific information.
- Transfer learning: after the pretrained CNN is trained on the base dataset, it can be adapted for other tasks with smaller datasets or different domains. This process is called transfer learning. Instead of starting from scratch and training a deep neural network from the ground up, you can take advantage of the features and knowledge the CNN has already learned.
- Fine‐tuning: in transfer learning, you can adjust the weights of the pretrained CNN by training it on your specific task or dataset. This is often done by replacing the final classification layer(s) with new layers suited to your task and training only these layers while keeping the rest of the network's parameters frozen. This allows the model to adapt to the new task while retaining the general knowledge it gained from the base dataset.
- Data augmentation: in the context of pretrained CNNs, it refers to the technique of artificially increasing the size of a training dataset by applying various transformations (rotation, translation, scaling, flip, etc.) to the existing data. It is generally useful when there is a limited amount of training data. Nevertheless, earlier investigations utilizing subsets of the same IHC data did not find any improvements in pretrained models when employing data augmentation.^18^, ^41^ This study, therefore, does not incorporate data augmentation techniques.

Pretrained CNNs are useful for the purpose in this study, because they offer several benefits as follows.

- Reduced training time: training deep neural networks from scratch can be computationally expensive and time‐consuming. Using pretrained models as a starting point reduces both time and computational resources.
- Improved performance: pretrained CNNs have already learned to extract useful features from images. Fine‐tuning these models on the specific task of learning IHC images often leads to better performance compared to training from scratch, especially when the IHC data size are limited.
- Generalization: pretrained CNNs capture a broad range of visual features, which can help in tasks related to image understanding, even if the task is different from the original training task.
- Availability: many pretrained CNN models are publicly available, making them accessible to the research community.

In this study, 10 popular pretrained CNN models are adopted as baseline deep networks for predicting survival time of the rectal patient cohort. These pretrained models are briefly described as follows.

### 2.3 GoogLeNet

This CNN is a deep net, comprising 22 layers, and it operates on images with a resolution of 224 × 224 × 3 pixels.^42^ GoogLeNet employs a distinctive approach by incorporating multiple convolutional filter sizes within each module of the network, occasionally incorporating max‐pooling layers. The network was trained using a big dataset of over one million images sourced from the ImageNet database,^43^ with the goal of classifying these images into 1000 distinct object categories. For the purpose of feature extraction from IHC images in this study, the layer known as “pool5‐7x7_s1” (global average pooling) was specifically chosen.

### 2.4 AlexNet

This is a CNN of eight layers deep, including five convolutional layers with occasional max‐pooling layers and three fully connected layers with a final 1000‐way softmax, and has an image input size of 227 × 227 × 3.^44^ This net was trained with more than one million images of the ImageNet database^43^ to classify 1.2 million images into 1000 object categories. Layer “pool5” was chosen for the deep‐learning feature extraction of the IHC images.

### 2.5 ShuffleNet

This particular network is a CNN characterized by its depth of 50 layers and tailored to accommodate an image input size of 224 × 224 × 3 pixels.^45^ It was conceptualized and structured for effective deployment on mobile devices, where computational resources tend to be constrained. The architecture of this network revolves around two distinctive operations: pointwise group convolution and channel shuffling, both were designed to optimize classification accuracy while aligning with the computational limitations of mobile devices. In this study, the layer denoted as “node 200” was chosen to facilitate the extraction of deep‐learning features from IIHC images.

### 2.6 ResNet101

This deep neural network belongs to the residual networks (ResNet) family.^46^ It was pretrained and requires input images of 224 × 224 × 3 pixels. What sets this network apart is its ability to learn residual functions concerning the layer inputs, as opposed to the raw signals, and it achieves this by stacking residual blocks to create a network with a depth of 101 layers. This architectural innovation simplifies the optimization process, making it comparatively more straightforward to enhance the network performance by increasing its depth. In the context of this study, the “pool5” layer from ResNet101 was chosen to extract deep features from the IHC images.

### 2.7 DenseNet201

This network is a variation of the DenseNet model,^47^ specifically known as DenseNet‐201 due to its extensive depth of 201 layers. What distinguishes this architecture is its unique design, enabling the assimilation of collective information from preceding layers to reduce channel redundancy, resulting in a densely connected network. DenseNet‐201 requires images sized at 224 × 224 × 3 pixels. In this study, the “avg_pool” layer, which is the global average pooling, was selected to facilitate the extraction of deep features from IHC images.

### 2.8 SqueezeNet

SqueezeNet is a CNN characterized by its 18 layers, with an image input size of 227 × 227 × 3 pixels.^48^ This network underwent extensive training on a dataset exceeding one million images from the ImageNet database,^43^ where its objective was to categorize images into 1000 distinct object categories. Consequently, this network possesses a wealth of knowledge derived from a diverse range of images. For the purpose of this study involving IHC data analysis, the “pool10” layer (global average pooling) was chosen to extract deep features.

### 2.9 Xception

The nomenclature of this deep net signifies an advanced Inception model.^49^ Through enhancements to depth‐wise separable convolution, this network surpasses the performance of the Inception model. With a depth of 71 layers, it requires input images sized at 299 × 299 × 3 pixels. Its training was conducted using an extensive dataset of over a million images sourced from the ImageNet database,^43^ geared toward classifying these images into 1000 object categories. In this particular study focusing on IHC data analysis, the “avg_pool” layer was selected to facilitate the extraction of deep features.

### 2.10 InceptionResNetv2

This CNN has an impressive depth of 164 layers and requires input images sized at 299 × 299 × 3 pixels.^50^ InceptionResNetv2 is rooted in the Inception family, where it innovatively introduces residual connections by replacing the filter concatenation stage found in the Inception design. For the purposes of extracting deep features from IHC data in this study, the “avg_pool” layer was employed.

### 2.11 DarkNet53

This deep net^51^ serves as the foundational CNN architecture for the YOLOv3 object detection methodology.^52^ This network spans 53 layers in depth and was designed to handle images with a size of 256 × 256 × 3 pixels. Having been pretrained on a diverse dataset comprising over a million images from ImageNet,^43^ this CNN inherits a wealth of feature learning. In the context of this study involving IHC data analysis, the “avg1” layer was chosen for the extraction of deep features.

### 2.12 NASNetLarge

This network is a specialized iteration of the Neural Architecture Search Network (NASNet) models.^53^ It was crafted to incorporate both normal and reduction cells, facilitating the exploration of search space, search strategy, and performance evaluation to attain optimal performance. NASNet‐Large expects input images sized at 331 × 331 × 3 pixels. For the purpose of extracting deep features from IHC images in this study, the “global_average_pooling2d_2” layer of the NASNetLarge was employed.

### 2.13 Wavelet scattering network analysis of deep‐learning features

**FIGURE 1:**
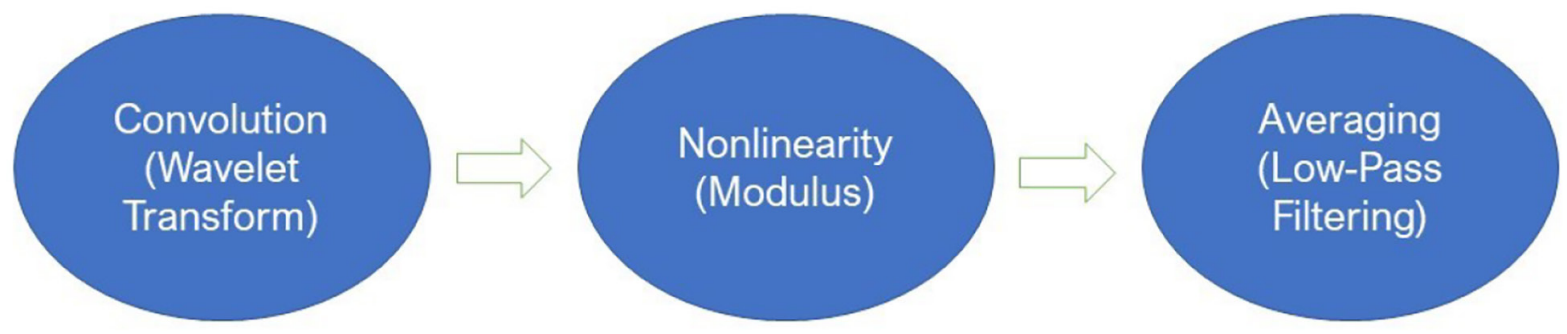
Three basic operations of a wavelet scattering process at each network layer.

The similarity between a signal and wavelets of varying frequency and scale at a time point can be measured with the continuous wavelet transform (CWT). The wavelet scattering network^54^ makes use of the CWT to perform three basic operations for the decomposition of a signal (see Figure 1): convolution, nonlinearity, and average pooling. These operations alter the original signal across a series of layers organized in a tree‐like structure, where the input for each subsequent layer stems from the output of its predecessor. The scattering process generates wavelet scattering coefficients at each layer of the network.

In this study, input signals for the wavelet scattering decomposition are the flattened feature vectors of the IHC images extracted by one of the described pretrained CNNs. The process of computing wavelet scattering coefficients iteratively across multiple layers is outlined as follows.

Let $\psi \left(t\right)$ be a band‐pass filter, which is known as a mother wavelet, and $\psi_{\lambda_{j}}\left(t\right)$ be a wavelet filter bank, which can be formed by expanding the mother wavelet as

(1) $$\psi_{\lambda_{j}}\left(t\right)=\lambda_{j}\psi \left(\lambda_{j}t\right),$$

where $\lambda_{j}=2^{\left(j/Q\right)}$, j∈ℤ, 1≤j≤J, *J* is the maximum level of layers, and *Q* is the number of wavelets per octave.

Let **f** be an input signal, which is a (flattened) deep‐learning feature vector of an input IHC image. The wavelet scattering coefficients at layer zero, which are also called the zeroth order scattering coefficients and computed by taking the average of the feature vector as

(2) $$L_{0}\left(t\right)=f^{*}\phi \left(t\right),$$

where $L_{0}$ denotes the zeroth order scattering, ϕ is a low‐pass filter, and * denotes the convolution operator.

Coefficients of the wavelet scattering at layer 1 or the first‐order wavelet scattering are determined by averaging the modulus of wavelet coefficients at Layer 1 as

(3) $$L_{1}\left(t,\lambda_{1}\right)=|f^{*}\psi_{\lambda_{1}}\left(t\right)|*\phi \left(t\right).$$

The second‐order wavelet scattering coefficients are calculated as

(4) $$L_{2}\left(t,\lambda_{1},\lambda_{2}\right)=\|f^{*}\psi_{\lambda_{1}}\left(t\right)|*\psi_{\lambda_{2}}\left(t\right)|*\phi \left(t\right).$$

Likewise, computation of the third‐order wavelet scattering coefficients is given as

(5) $$L_{3}\left(t,\lambda_{1},\lambda_{2},\lambda_{3}\right)=|\|f^{*}\psi_{\lambda_{1}}\left(t\right)|*\psi_{\lambda_{2}}\left(t\right)|*\psi_{\lambda_{3}}\left(t\right)|*\phi \left(t\right).$$

Typically, wavelet scattering coefficients at layers *j*, where j=1…J, are computed through the use of convolution, modulus, and average pooling operators, as follows

(6) $$L_{j}\left(t,\lambda_{1},\ldots ,\lambda_{j}\right)=|\ldots \|f^{*}\psi_{\lambda_{1}}\left(t\right)|{\;}^{*}\psi_{\lambda_{2}}\left(t\right)|\ldots *\psi_{\lambda_{j}}\left(t\right)|*\phi \left(t\right).$$

The Morlet wavelet, which is widely used for wavelet scattering, is adopted as the mother wavelet and defined as.^55^, ^56^

(7) $$\psi \left(t\right)=c\left[e^{-\frac{t^{2}}{2\sigma^{2}}}\right]e^{2\text{πift}},$$

where, in this study, *c* = 1, σ=1, *i* is the imaginary unit, *f* is the center frequency, and 2πf=5, and $e^{2\text{πift}}=\cos\left(5t\right)$, yielding $\psi \left(t\right)=e^{\frac{t^{2}}{2}}\cos\left(5t\right)$.

Other parameters for wavelet scattering in this study were specified as follows. Scale of time invariance = half of signal length, using wavelet scattering network with two filter banks, where Q factor for filter bank 1 = 8 wavelets per octave, and *Q* factor for filter bank 2 = 1 wavelet per octave, and sampling frequency = 1 Hz.

The mathematical operations underpinning wavelet scattering, as explained earlier, prove valuable in the context of texture feature extraction from IHC images within the domain of pattern classification. This transform presents several advantages for such an application, such as,

- Multiscale analysis: wavelet scattering transform operates at multiple scales, allowing it to capture both fine and coarse texture information within an image. Textural patterns often exhibit variations at different scales, and the ability to analyze these scales simultaneously is crucial for robust feature extraction.
- Translation invariance: wavelet scattering transform provides translation invariance, which means it can identify and describe textural patterns regardless of their position in the image. In textural analysis, patterns may appear in different locations, and translation invariance ensures that the extracted features are not sensitive to spatial shifts.
- Rotation invariance: in some cases, textural patterns can appear at different orientations. Wavelet scattering transform can be extended to provide rotation invariance, making it adaptable to a wider range of textural variations.
- Hierarchical features: the transform provides a hierarchical representation of the image, capturing both low‐level and high‐level features. This hierarchical feature representation can be particularly valuable for pattern classification tasks where different levels of abstraction are required.
- State‐of‐the‐art performance: empirical studies have shown that wavelet scattering transform often outperforms traditional texture feature extraction methods, such as gray‐level co‐occurrence matrix and local binary patterns, in various pattern classification tasks.^57^, ^58^, ^59^

### 2.14 Support vector machines

A model of support vector machines (SVMs)^60^, ^61^ adopted in this study is the linear SVM. It is a supervised machine‐learning algorithm used for binary classification tasks. Its primary objective is to find a hyperplane that best separates two classes of data points in a feature space. The key characteristics and components of a linear SVM are described as follows.

- Hyperplane: in a two‐dimensional feature space, a hyperplane is a straight line that separates two classes of data points. In feature spaces with more than two dimensions, a hyperplane can be described as a flat affine subspace whose dimension is one less than that of the surrounding space. For binary classification, the hyperplane is used to distinguish between the two classes.
- Margin: the margin is the distance between the hyperplane and the nearest data point from either class. In linear SVM, the goal is to find the hyperplane that maximizes this margin. A larger margin generally indicates better generalization performance.
- Support vectors: support vectors are the data points that are closest to the hyperplane and play a crucial role in defining the margin. These are the points that, if moved or altered, would affect the position of the hyperplane.
- Linear separability: linear SVM works well when the data are linearly separable, meaning that a hyperplane can completely separate the two classes without any misclassifications.
- Soft margin: in situations where the data are not perfectly separable by a hyperplane, linear SVM can be adapted to allow for some misclassifications. This is done by introducing a soft margin that allows some data points to be on the wrong side of the hyperplane while still aiming to minimize misclassifications and maximize the margin.
- Kernel trick: linear SVM can be extended to handle nonlinearly separable data by using a technique called the kernel trick. This involves mapping the original feature space into a higher dimensional space, where the data may become linearly separable. Common kernel functions include polynomial kernels and radial basis function kernels.
- Optimization: the training of a linear SVM involves solving a convex optimization problem to find the optimal hyperplane that maximizes the margin while minimizing classification errors (soft margin case). This optimization is typically achieved using methods like the sequential minimal optimization algorithm.
- Regularization: linear SVM can incorporate regularization terms to prevent overfitting. Regularization helps in controlling the complexity of the model and is especially useful when dealing with high‐dimensional data.
- Decision boundary: the decision boundary of a linear SVM is defined by the hyperplane. Data points on one side of the hyperplane are classified as one class, while data points on the other side are classified as the other class.
- Classification: once trained, the linear SVM can be used to classify new data points by determining on which side of the hyperplane they fall.

Hereafter, the term SVM shall denote the linear SVM.

### 2.15 Double convolutional features for SVM‐based predictor

Having described the selected pretrained CNN models for extracting deep features from the protein‐expressed IHC images obtained from cohorts of > and <5‐year survival times, and wavelet scattering network analysis as another convolutional feature extractor of the deep‐learning features, training, and testing phases of the proposed predictor are designed as follows.

For training a classifier, the 10‐fold cross‐validation was used, where nine randomly selected folds of the IHC images of each of the two survival classes were input into a pretrained CNN for transfer learning. The flattened learned feature vectors were extracted at the selected deep layer of the network as previously specified in the descriptions of the 10 pretrained CNN models. These convolutional feature vectors were then used as input signals for the wavelet scattering transform, resulting in the extraction of wavelet scattering coefficients or second convolutional features from the training IHC images. Finally, the extracted wavelet scattering features were used for training an SVM‐based classifier for predicting the survival time. In this study, parameters specified for the SVM model are: kernel function = polynomial of order 2, kernel scale = 1, solver = sequential minimal optimization, and data = standardized.

For testing the trained classifier, the other 10th folds of the IHC images of the two survival classes were used for extracting the wavelet scattering coefficients following the same procedure applied in the training phase. The wavelet scattering coefficients extracted from each image of the test data were then feed into the trained SVM‐based classifier for the survival time prediction.

**FIGURE 2:**
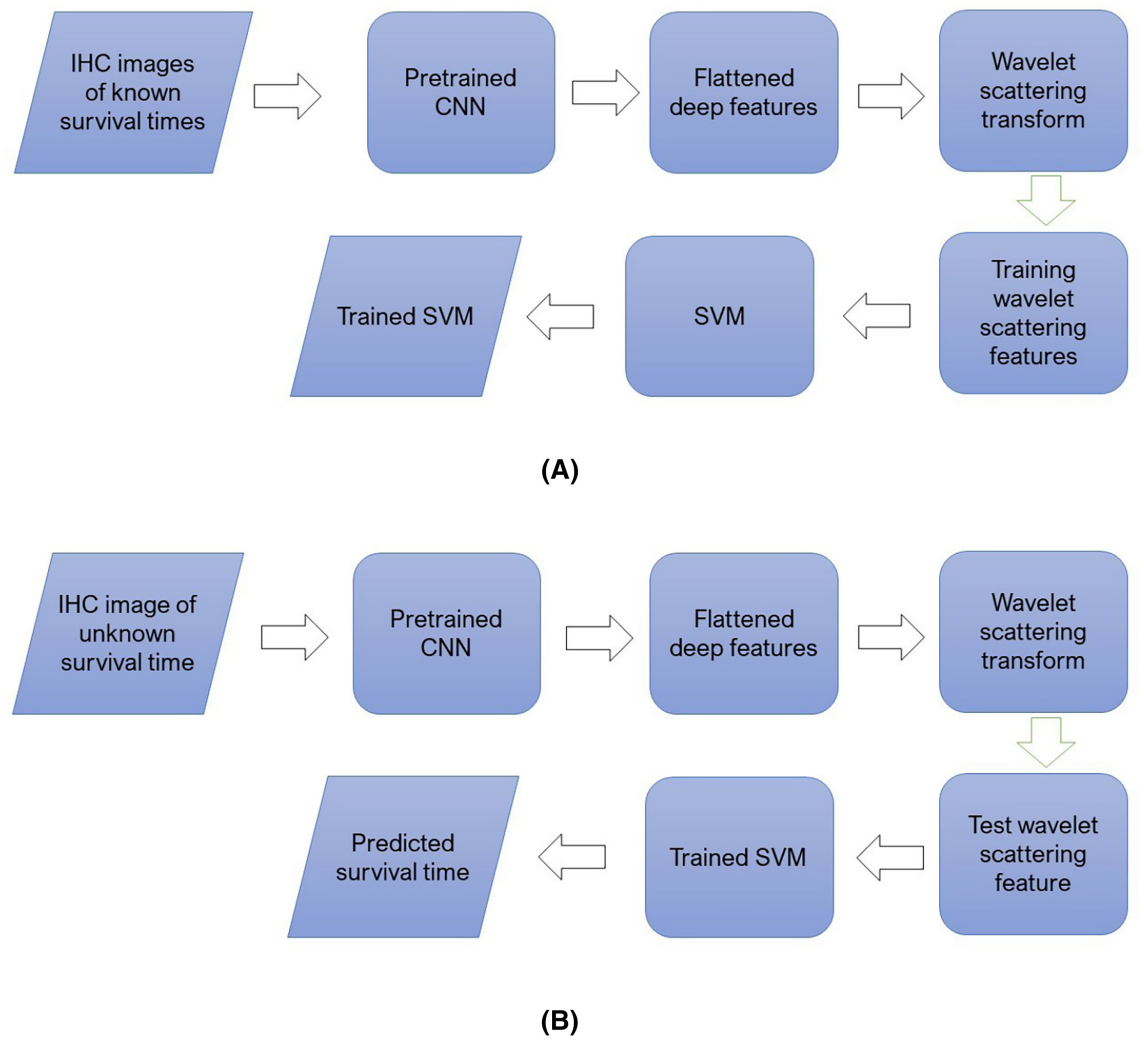
Training (A) and testing (B) phases of the proposed approach.

The above processes of training and testing were repeated 10 times to ensure each of the 10 folds was tested. Thus, the validation resulted in a final confusion matrix accumulated from the 10 runs of training and testing of the whole protein‐expressed IHC data for each of the tissue types. Figure 2 graphically describes the training and testing phases of the proposed approach. During the training phase, multiple IHC images (either RhoB or DNp73) with known survival times are used to extract wavelet‐scattering features, facilitating the training of the SVM model. This results in an SVM model that has been trained to predict two classes: survival times greater than 5 years and survival times less than 5 years. In the subsequent testing phase, an IHC image of either RhoB or DNp73 expression, for which the survival time is unknown, is initially processed through a pretrained CNN for deep feature extraction. These features are then converted into wavelet scattering coefficients, which serve as input data for the trained SVM model. This trained SVM model is then employed to predict the survival time of the patient with rectal cancer.

### 2.16 Measures of prediction performance

The following variables are defined as

- *P* = the count of samples of less than 5‐year survival
- *N* = the count of samples of greater than 5‐year survival
- *TP* = the count of correctly predicted samples of less than 5‐year survival
- *TN* = the count of correctly predicted samples of greater than 5‐year survival
- *FP* = the count of wrongly predicted samples of less than 5‐year survival
- *FN* = the count of wrongly predicted samples of greater than 5‐year survival

Prediction accuracy (*ACC*), correct detection for <5‐year survival time (*D*(<5)), correct detection for >5‐year survival time (*D*(>5)), precision (*PRC*), and *F*_1_ score, which are used as statistical measures of predictor performance, are defined as follows.

(8) $$\mathrm{ACC}=\frac{\mathrm{TP}+\mathrm{TN}}{P+N}.$$

(9) $$D\left(<5\right)=\frac{\mathrm{TP}}{P}.$$

(10) $$D\left(>5\right)=\frac{\mathrm{TN}}{N}.$$

(11) $$\mathrm{PRC}=\frac{\mathrm{TP}}{\mathrm{TP}+\mathrm{FP}}.$$

(12) $$F_{1}=\frac{2\mathrm{TP}}{2\mathrm{TP}+\mathrm{FP}+\mathrm{FN}}.$$

Another performance measure is the area under the receiver operating characteristic (ROC) curve. The ROC curve is constructed by plotting the TP rate against the FP rate at various thresholds. In other cases, the TP rate is called sensitivity or the probability of prediction and the FP rate is also known as the probability of false alarm. Thus, the area under the ROC curve (AUC) measures the quality of a predictor regardless what classification threshold is chosen.

Higher values of the measures expressed in Equations ((8), (9), (10), (11), (12)) and the AUC indicate better performance of the predictor.

## 3 RESULTS

### 3.1 Five‐year survival prediction with RhoB expression

The RhoB‐expressed IHC images of tumors obtained from patients with RT were used to illustrate the robustness and significant reduction in computing speed of the proposed combination approach. Table 1 shows the performance measures between the 10 pretrained CNNs, which extracted deep‐learning features of the IHC images and predicted the survival time; and the 10 proposed methods, which used the pretrained CNNs as the first convolutional learning for extracting deep‐learning features of the IHC images, WS as the second convolutional learning for extracting multilevel‐filtered features from the deep‐learning features, and SVM for predicting the survival time. Results obtained from the 10‐fold cross‐validation include accuracy, predictions for < and >5 years, precision, *F*_1_ score, AUC, and total computing time using a single GPU (Quadro RTX 6000).

**TABLE 1:** Ten‐fold cross‐validations of 5‐year survival prediction using RhoB expression in rectal cancer tumor tissue with RT: comparative analysis.

Model	*ACC*	*D*(<5)	*D*(>5)	*PRC*	*F*_1_	AUC	GPU time (s)
Pretrained CNNs (Baseline)							
InceptionResNetv2	57.55	92.45	22.64	75.00	0.35	0.57	9417
NASNetLarge	57.55	62.26	52.83	58.33	0.55	0.59	12,039
ResNet101	58.49	58.49	58.49	58.49	0.58	0.63	9818
GoogLeNet	61.32	60.38	62.26	61.11	0.62	0.68	9721
Xception	61.32	54.72	67.92	60.00	0.64	0.63	9370
AlexNet	62.26	58.49	66.04	61.40	0.64	0.65	9691
DenseNet201	64.15	62.26	66.04	63.64	0.65	0.70	9481
DarkNet53	64.15	58.49	69.81	62.71	0.66	0.67	9352
ShuffleNet	66.04	60.38	71.70	64.41	0.68	0.68	9898
SqueezeNet	66.98	60.38	73.58	65.00	0.69	0.67	7915
Proposed approach							
DenseNet201‐WS‐SVM	79.25	89.58	87.23	89.13	0.88	0.81	21
SqueezeNet‐WS‐SVM	84.91	91.84	93.75	91.84	0.93	0.87	16
InceptionResNetv2‐WS‐SVM	84.91	91.30	97.96	92.31	0.95	0.89	24
DarkNet53‐WS‐SVM	89.62	96.23	93.62	95.65	0.95	0.92	18
GoogLeNet‐WS‐SVM	92.45	100	92.31	100	0.96	0.93	16
ShuffleNet‐WS‐SVM	93.40	98.11	88.68	97.92	0.93	0.91	18
Xception‐WS‐SVM	94.34	98.00	98.08	98.08	0.98	0.95	18
ResNet101‐WS‐SVM	94.34	98.00	100	98.08	0.99	0.94	20
NASNetLarge‐WS‐SVM	96.23	96.15	100	96.30	0.98	0.97	29
AlexNet‐WS‐SVM	97.17	98.11	96.23	98.08	0.97	0.96	18

Table 2 shows the 10‐fold cross‐validation classification results obtained from the top 3 models found in Table 1, which are ResNet101‐WS‐SVM, NASNetLarge‐WS‐SVM, and AlexNet‐WS‐SVM for the RhoB‐expressed IHC samples of tumor, biopsy, metastatic, and adjacent normal tissues with RT and non‐RT.

**TABLE 2:** Ten‐fold cross‐validations of 5‐year survival prediction using RhoB expression in rectal cancer, using top three proposed models.

Model	*ACC*	*D*(<5)	*D*(>5)	*PRC*	*F*_1_	AUC
With RT						
Tumor tissue						
ResNet101‐WS‐SVM	94.34	98.00	100	98.08	0.99	0.94
NASNetLarge‐WS‐SVM	96.23	96.15	100	96.30	0.98	0.97
AlexNet‐WS‐SVM	97.17	98.11	96.23	98.08	0.97	0.96
Biopsy tissue						
ResNet101‐WS‐SVM	98.39	100	100	100	1	0.96
AlexNet‐WS‐SVM	100	100	100	100	1	0.97
NASNetLarge‐WS‐SVM	100	100	100	100	1	0.99
Metastatic tissue						
ResNet101‐WS‐SVM	100	100	100	100	1	1
AlexNet‐WS‐SVM	100	100	100	100	1	1
NASNetLarge‐WS‐SVM	100	100	100	100	1	1
Adjacent normal tissue						
AlexNet‐WS‐SVM	98.28	96.55	100	96.67	0.98	0.98
NASNetLarge‐WS‐SVM	98.28	100	100	100	1	0.99
Xception‐WS‐SVM	100	100	100	100	1	1
Without RT						
Tumor tissue						
Xception‐WS‐SVM	93.94	96.88	96.88	96.88	0.97	0.94
NASNetLarge‐WS‐SVM	97.73	100	98.44	100	0.99	0.97
AlexNet‐WS‐SVM	98.48	98.48	98.48	98.48	0.98	0.94
Biopsy Tissue						
ResNet101‐WS‐SVM	97.78	100	97.73	100	0.99	0.96
NASNetLarge‐WS‐SVM	98.89	100	97.78	100	0.99	0.98
AlexNet‐WS‐SVM	100	100	100	100	1	0.99
Metastatic tissue						
ResNet101‐WS‐SVM	100	100	100	100	1	1
NASNetLarge‐WS‐SVM	100	100	100	100	1	1
AlexNet‐WS‐SVM	100	100	100	100	1	1
Adjacent normal tissue						
Xception‐WS‐SVM	98.68	100	100	100	1	0.97
ShuffleNet‐WS‐SVM	100	100	100	100	1	0.93
AlexNet‐WS‐SVM	100	100	100	100	1	0.99

**FIGURE 3:**
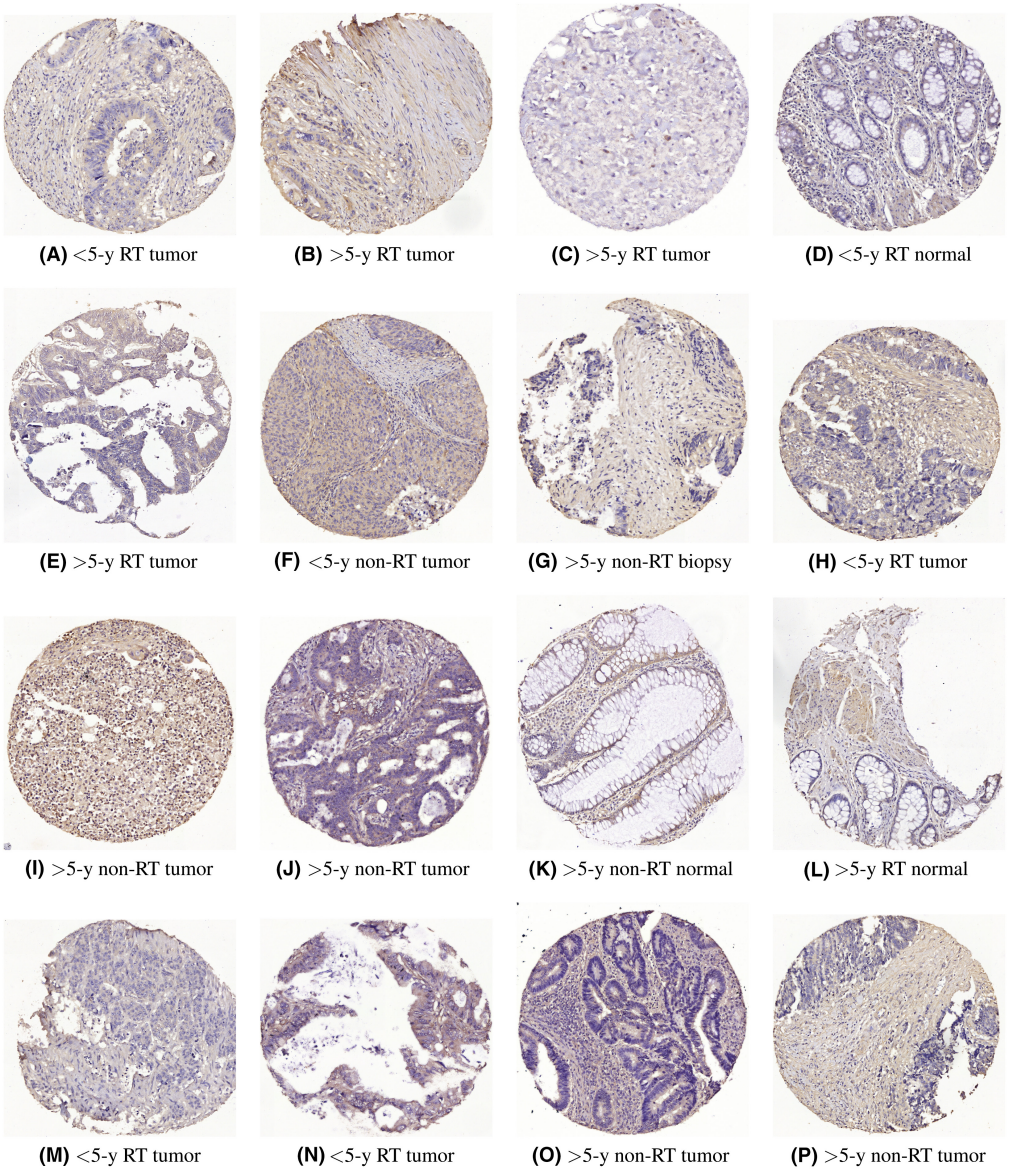
RhoB‐expressed samples of survival time of less (<5‐y) or more (>5‐y) than 5 years: falsely predicted by AlexNet‐WS‐SVM (A–F); falsely predicted by NASNetLarge‐WS‐SVM (G–K); and decided as tie votes by NASNetLarge‐WS‐SVM (L–P). Survival times of these images could not also be determined by two pathologists.

Figure 3 presents the RhoB‐expressed IHC images that were incorrectly classified or tied by the predictors listed in Table 2.

### 3.2 Five‐year survival prediction with DNp73 expression

**FIGURE 4:**
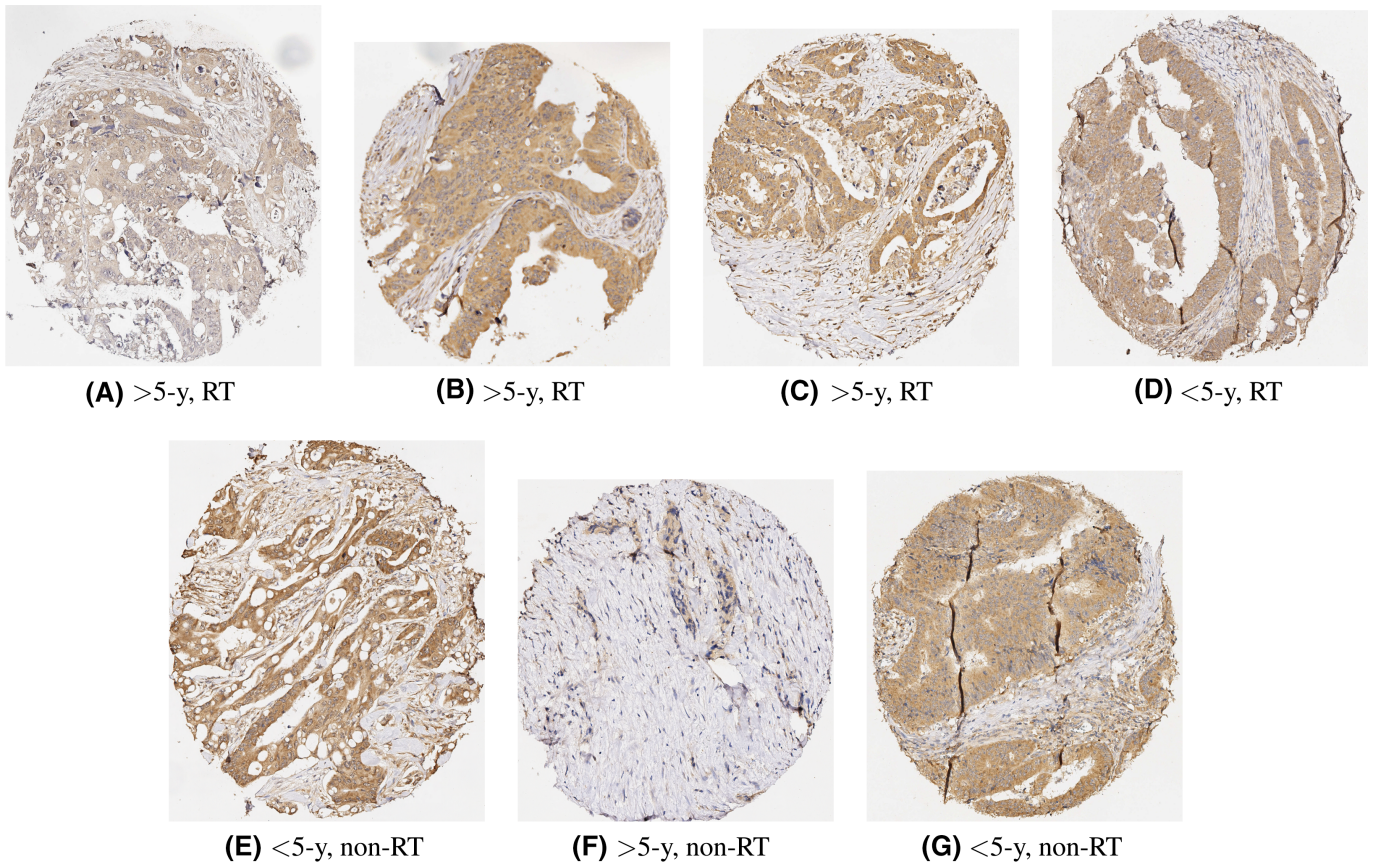
DNp73‐expressed tumor samples of survival time of less (<5‐y) or more (>5‐y) than 5 years: falsely predicted by ShuffleNet‐WS‐CNN (A–C); falsely predicted by ResNet101‐WS‐CNN (D); falsely predicted by AlexNet‐WS‐CNN (E); decided as tie votes by Xception‐WS‐CNN (F) and NASNetLarge‐WS‐CNN (G). Survival times of these samples could not also be determined by two pathologists.

Table 3 shows the 10‐fold cross‐validation classification results obtained from the top five models found in Table 1, which are ShuffleNet‐WS‐SVM, Xception‐WS‐SVM, ResNet101‐WS‐SVM, NASNetLarge‐WS‐SVM, and AlexNet‐WS‐SVM for the DNp73‐expressed IHC samples of tumor tissue with RT and non‐RT. Figure 4 presents the DNp73‐expressed IHC images that were incorrectly classified or tied by the predictors listed in Table 2.

**TABLE 3:** Ten‐fold cross‐validations of survival prediction using DNp73 expression in rectal cancer, using top five proposed models.

Model	*ACC*	*D*(<5)	*D*(>5)	*PRC*	*F*_1_	AUC
With RT						
Tumor tissue						
ShuffleNet‐WS‐SVM	93.48	100	86.96	100	0.93	0.93
ResNet101‐WS‐SVM	97.83	95.65	100	95.83	0.98	0.97
Xception‐WS‐SVM	100	100	100	100	1	0.99
NASNetLarge‐WS‐SVM	100	100	100	100	1	0.99
AlexNet‐WS‐SVM	100	100	100	100	1	0.99
Biopsy Tissue						
ShuffleNet‐WS‐SVM	100	100	100	100	1	0.98
Xception‐WS‐SVM	100	100	100	100	1	1
ResNet101‐WS‐SVM	100	100	100	100	1	1
NASNetLarge‐WS‐SVM	100	100	100	100	1	1
AlexNet‐WS‐SVM	100	100	100	100	1	1
Without RT						
Tumor tissue						
AlexNet‐WS‐SVM	98.00	96.00	100	96.15	0.98	0.99
Xception‐WS‐SVM	98.00	100	100	100	1	0.99
ShuffleNet‐WS‐SVM	100	100	100	100	1	0.94
ResNet101‐WS‐SVM	100	100	100	100	1	0.99
NASNetLarge‐WS‐SVM	98.00	100	100	100	1	0.99
Biopsy tissue						
ShuffleNet‐WS‐SVM	100	100	100	100	1	0.98
ResNet101‐WS‐SVM	100	100	100	100	1	0.99
Xception‐WS‐SVM	100	100	100	100	1	1
NASNetLarge‐WS‐SVM	100	100	100	100	1	1
AlexNet‐WS‐SVM	100	100	100	100	1	1

## 4 DISCUSSION

For the prediction of survival time using RhoB expression on the tumor tissue of RT patients, accuracy rates obtained from the 10 individual CNNs are between 58% (InceptioResNetv2) and 67% (SqueezeNet) with GPU times of 9417 and 7915 seconds, respectively. Using the same data, the lowest and highest accuracies obtained from the proposed approach are 79% (DenseNet201‐WS‐SVM) and 97% (AlexNet‐WS‐SVM) with GPU times of 21 and 18 seconds. In comparison between the two best results obtained from the two approaches, the accuracy and computing time reduction provided by the proposed approach are 30% (97% vs. 67%) higher and 440 times (7915/18 seconds) faster than the baseline approach, respectively. Values of the AUC obtained from the 10 baseline methods are between 0.57 (InceptionResNetv2) and 0.70 (DenseNet201). Values of the AUC obtained from the 10 proposed methods are between 0.81 (DenseNet201‐WS‐SVM) and 0.97 (NASNetLarge‐WS‐SVM). For the two best proposed models, NASNetLarge‐WS‐SVM predicted >5‐year survival (100%) better than <5‐year survival (96%), and the other way around is for AlexNet‐WS‐SVM (96% and 98% for > and <5 years, respectively). The precision provided by AlexNet‐WS‐SVM (98%) is higher than that by the NASNetLage‐WS‐SVM (96%). *F*_1_ score, which is the harmonic mean of precision and <5‐year survival, obtained from NASNetLage‐WS‐SVM is slightly higher than AlexNet‐WS‐SVM (98% vs. 97%). A computing advantage for the use of AlexNet over NASNetLage is that AlexNet has smaller memory size (227 MB vs. 332 MB) and less model parameters ($61\times 10^{6}$ vs. $88.9\times 10^{6}$) than NASNetLarge. Not only this comparative analysis shows the high performance of the proposed approach but also its significant improvement over the baseline CNNs in terms of prediction accuracy and computational time.

The top three proposed models identified from Table 1 were selected for the survival time prediction of RT and non‐RT patients using other types of tissue captured with RhoB‐expressed IHC images, which are presented in Table 2. For the RT case, both AlexNet‐WS‐SVM and NASNetLarge‐WS‐SVM perfectly predicted the survival time (100%) using either the biopsy or metastatic samples, while the Xception‐WS‐SVM perfectly predicted the survival time (100%) using samples of normal tissue adjacent to tumors. For the non‐RT case, AlexNet‐WS‐SVM appears to be the best prediction model using samples of tumor (98%), biopsy (100%), metastatic (100%), and normal (100%) tissues. For both RT and non‐RT cases, the double convolutional learning of metastatic samples appears to be the most favorable for the survival prediction task (100% accuracy obtained from all three predictors).

The top five proposed models found in Table 1 were selected for the survival time prediction of RT and non‐RT patients using DNp73‐expressed IHC images of tumor and biopsy tissues, which are shown in Table 3. Being similar to the prediction using RhoB expression, two most robust models in terms of accuracy and AUC measures are AlexNet‐WS‐SVM and NASNetLarge‐WS‐SVM. All five proposed models perfectly predicted the survival time using the IHC images of biopsy (100%). Three models that perfectly predicted the survival time using the IHC images of tumor with RT (100%) are Xception‐WS‐SVM, NASNetLarge‐WS‐SVM, and AlexNet‐WS‐SVM. Two models that perfectly predicted the survival time using the IHC images of tumor without RT (100%) are ShuffleNet‐WS‐SVM, and ResNet101‐WS‐SVM. By taking tie votes into account, predictions for either < or >5 years, including with and without RT, were robustly performed by all five models (96%–100%), except for ShuffleNet‐WS‐SVM that yielded 87% for the >5‐year survival using the tumor samples with RT. All five models achieved high precision rates (96%–100%), high F1 scores (0.93–1), and high AUC values (0.93–1). Once again, the validation results in terms of accuracy, specific survival time predictions, precision, *F*_1_ score, and AUC demonstrate the potential application of the double convolutional learning for discovering the predictive power of protein DNp73 in rectal cancer.

Robustness of the proposed approach is further demonstrated by its learning on relatively very limited data in the case of DNp73 expression, where there are small number of images for each survival group. Using the 10‐fold cross‐validation, for the RT case, the accuracies are very high (93%–100%) for the survival time classification using the tumor tissue (100%), and biopsy tissue (100%). Likewise, using the 10‐fold cross‐validation for the RT case, the accuracies are even higher (98%–100%) for the survival time classification using the tumor tissue (100%), and biopsy tissue (100%).

Figures 3 and 4 show IHC images of proteins RhoB and DNP73 expressions, respectively, which were mistaken or tied by certain classifiers of the proposed approach. As indicated in Tables 1, 2, 3, most images that failed the prediction are of the tumor tissue, and in particular, RT tumor samples (4 for < and 3 for >5 years = 7 out of 12 for RhoB expression, and 1 for < and 3 for >5 years = 4 out 7 for DNp73 expression). However, two pathologists at Linkoping University could not determine the survival times of these images that can be separated for further examination to gain insight into assessing, labeling, and visualizing the protein levels of gene expression of the targeted detection reagents.

Additionally, Table 4 presents a comparison of the top three prediction accuracies achieved through the utilization of RhoB and DNp73 expressions. This analysis indicates that DNp73 expression has the potential to offer more favorable prognostic insights compared to RhoB when employing AI for the analysis of both tumor and biopsy tissues, with or without RT. Among the models evaluated, Xception‐WS‐SVM emerges as the preferred choice for learning from DNp73 expression data, while AlexNet‐WS‐SVM exhibits superior performance when learning from RhoB expression data.

**TABLE 4:** Top three accuracies (%) obtained from 10‐fold cross‐validations of 5‐year survival prediction using RhoB and DNp73 expressions in rectal cancer.

With RT	
Tumor tissue	
RhoB	DNp73
94.34 (ResNet101‐WS‐SVM)	100 (Xception‐WS‐SVM)
96.23 (NASNetLarge‐WS‐SVM)	100 (NASNetLarge‐WS‐SVM)
97.17 (AlexNet‐WS‐SVM)	100 (AlexNet‐WS‐SVM)
Biopsy tissue	
RhoB	DNp73
98.39 (ResNet101‐WS‐SVM)	100 (Xception‐WS‐SVM)
100 (AlexNet‐WS‐SVM)	100 (NASNetLarge‐WS‐SVM)
100 (NASNetLarge‐WS‐SVM)	100 (AlexNet‐WS‐SVM)
Without RT	
Tumor tissue	
RhoB	DNp73
93.94 (Xception‐WS‐SVM)	98.00 (Xception‐WS‐SVM)
97.73 (NASNetLarge‐WS‐SVM)	100 (ShuffleNet‐WS‐SVM)
98.48 (AlexNet‐WS‐SVM)	100 (ResNet101‐WS‐SVM)
Biopsy tissue	
RhoB	DNp73
97.78 (ResNet101‐WS‐SVM)	100 (Xception‐WS‐SVM)
98.89 (NASNetLarge‐WS‐SVM)	100 (NASNetLarge‐WS‐SVM)
100 (AlexNet‐WS‐SVM)	100 (AlexNet‐WS‐SVM)

The choice of using specific protein markers for cancer prognosis in research or clinical practice can depend on several factors. Some reasons why this study focused on two protein markers RhoB^10^, ^62^ and DNp73^63^, ^64^ are because of their relevance to this cancer type (different cancers can have distinct molecular profiles, and specific protein markers may be more relevant to one type of cancer than another), biological significance (RhoB and DNp73 have known roles in colorectal cancer progression, making them of interest for prognosis studies), and hypothesis testing (RhoB and DNp73 are hypothesized to have valuable prognostic information for colorectal cancer). Conducting experiments or assays to measure protein markers can be resource‐intensive and time‐consuming. Therefore, this report limits the number of markers to ensure the feasibility of the study.

Cancer is a complex disease with numerous variables that can affect survival time. While protein markers can be important biomarkers for predicting outcomes, they are just one part of a comprehensive assessment that considers the patient's overall health, the characteristics of the cancer itself, and the effectiveness of treatment. Nonetheless, there are instances where patients' profiles do not yield valuable insights for cancer prognosis. In particular, the characteristics of these rectal cancer patients do not reveal useful information for prognosis. This can be due to several possible reasons, including (1) sample size: a small sample size may not be representative of the broader population of rectal cancer patients, making it challenging to draw statistically significant conclusions about prognosis; (2) heterogeneity: rectal cancer is a heterogeneous disease, and patients can present with various clinical and pathological features; and (3) variability in disease progression: the course of rectal cancer can vary widely from one patient to another, and clinical and pathological characteristics alone may not encompass all the relevant factors influencing prognosis.

As overfitting is a common problem in machine learning, particularly in the context of supervised learning, where a model is trained to predict a target variable based on input data. Overfitting occurs when a machine‐learning model learns the training data too well, to the extent that it starts to memorize the noise and random fluctuations in the training data, rather than learning the underlying patterns. As a result, an overfit model performs very well on the training data but poorly on unseen or new data, which is the data it have not encountered during training.

Although the 10‐fold cross‐validation is a commonly used and effective method to mitigate overfitting and assess the performance of a machine‐learning model, it is worth noting that there are variations of cross‐validation, such as stratified k‐fold cross‐validation, which ensures that each fold preserves the class distribution of the original dataset. Stratified k‐fold is particularly useful when dealing with imbalanced datasets. In this study, as mentioned earlier, to address the issue of data imbalance in machine learning and ensure an equitable representation of protein expression discoveries, the quantities of IHC images for RhoB and DNp73 expressions, for both survival time categories, were adjusted to achieve equal distributions. This adjustment involves reducing the number of images in the majority class to match that of the minority class. In spite of this strategy for achieving data balance, to confirm the robustness of the proposed approach, specific patient‐independent data were used as test sets to predict survival times, utilizing samples of RhoB‐expressed metastatic tissue with and without RT, as well as DNp73‐expressed biopsy tissues with and without RT. The use of patient‐independent data achieved the same accuracies (100%) as those reported in Tables 2 and 3.

As another issue, ensemble methods in machine learning combine predictions from multiple data types or models to improve overall performance and robustness. While it is possible to use different tissue types (tumor, biopsy, metastasis, and adjacent normal tissue) together in an ensemble method to predict survival time, there are several challenges and considerations that may make it more common to analyze them separately. Some reasons for this separation to be preferred include: (1) biological differences (different tissue types may have distinct biological characteristics, gene expressions, and molecular profiles. Combining them in a single model might not capture these variations effectively), (2) data availability (tissue samples can vary widely in terms of data availability. For example, in this study, samples of tumor tissue may have more than biopsy, metastasis, and adjacent normal tissue. Combining data from various tissue types might not be compatible or introduce bias into the model), (3) heterogeneity (tumors are known for their intratumor heterogeneity, which means that different parts of the tumor may exhibit different genetic and molecular characteristics. Combining tumor, biopsy, metastasis, and normal tissue data can further increase this heterogeneity and complicate the feature extraction process), and (4) interpretability (combining different tissue types in a single model can make it challenging to interpret the contributions of each tissue type to the prediction. Understanding which tissue type is more informative for survival prediction can be essential for medical research).

In a previous study,^19^ various combinations of pretrained CNNs and SVM were explored for prognostic purposes in rectal cancer patients who had not received RT. These combinations included ResNet18‐SVM, SqueezeNet‐SVM, DenseNet201‐SVM, AlexNet‐SVM, Xception‐SVM, and NASNetLarge‐SVM, all of which utilized the same IHC data of RhoB‐expressed biopsy tissues. Among these combined models, NASNetLarge‐SVM exhibited the most promising results, achieving an accuracy of 85% in a 10‐fold cross‐validation setting. However, it is worth noting that the combined models incorporating pretrained CNNs, WS features, and SVM, as reported in this study, surpassed this accuracy level.

Another previous study^41^ focused on predicting the survival time of rectal‐cancer patients who had received RT. In this study, NASNetLarge‐SVM, DenseNet201‐SVM, and ResNet101‐SVM were applied using the same IHC data of normal tissues adjacent to tumors. Among these models, DenseNet201‐SVM achieved the highest 10‐fold cross‐validation accuracy of 75%. However, the combination of pretrained CNNs, WS features, and SVM yielded superior results compared to these models.

Furthermore, the study reported in^41^ introduced an alternative approach involving pretrained CNNs, fuzzy recurrence plots (FRPs),^65^ and long short‐term memory (LSTM) networks.^66^ This innovative combination demonstrated improved performance over the pretrained CNN‐SVM models. Nevertheless, even the best‐performing pretrained CNN‐FRP‐LSTM model, with ResNet101, achieved an accuracy of 88%, which is still lower than the CNN‐WS‐SVM models presented in our study.

In comparison with studies reported in,^18^, ^41^ not only the AI‐based approach developed in current work was tested with many types of the cancer tissues (biopsy, tumor, metastasis, adjacent normal) with RT and non‐RT, but also able to consistently achieve much higher prediction rates in all cases. In comparison between using the pretrained CNN and SVM models for classification, training of the SVM model with the double convolution‐based extracted features could provide a much faster computational speed than the pretrained CNNs. Such a saving in computing time makes the proposed approach economical and feasible for learning on big data as expected in our future acquisition or other applications.

## 5 CONCLUSION

An approach for extracting strongly differentiable protein‐expressed features from IHC images in rectal cancer by applying two different convolution‐based methods has been presented, validated, and discussed. Not only the proposed approach could achieve very high prediction rates but also relatively fast computing time. Furthermore, the proposed algorithm is effective for learning on small datasets, offering many advantages over more data‐intensive methods, where limited data exist, such as predicting treatment outcome or disease risk for a population whose electronic health records are not available.^67^

The results reported in this study are very encouraging for adopting and further exploring the combined AI algorithms of pretrained deep networks, multilevel analysis of wavelet scattering, and SVM‐based classification for discovering and validating rectal or other cancer biomarkers using IHC data.

## AUTHOR CONTRIBUTIONS

**Tuan Pham:** Conceptualization (lead); formal analysis (equal); investigation (equal); methodology (lead); software (lead); validation (equal); writing – original draft (lead); writing – review and editing (lead). **Xiao feng Sun:** Conceptualization (supporting); data curation (lead); formal analysis (equal); investigation (equal); validation (equal); writing – review and editing (supporting).

## CONFLICT OF INTEREST STATEMENT

The authors state that there are no conflicts of interest.

## ETHICS STATEMENT

This study reused the image data, which are publicly available. Therefore, the authors did not need to obtain ethical approval for this study.

## Data Availability

The reported results can be reproduced using the IHC imaging data and MATLAB codes implemented in this paper. Both data and Matlab codes are available at the author's personal homepage: https://sites.google.com/view/tuan‐d‐pham/codes under the titles “Double convolutional learning of protein expression in rectal cancer” for the Matlab codes, “Artificial intelligence‐based 5‐year‐survival prediction and prognosis of DNp73 expression in rectal cancer patients” for DNp73 data,^18^ and “Rectal cancer biopsy” for RhoB data.^19^
